# Supplementary material for: Construction of Development Scores to Analyze Inequalities in Childhood Immunization Coverage: A Global Analysis from 2000 to 2021
Source: Int J Environ Res Public Health. 2025 Jun 16;22(6):941. doi: 10.3390/ijerph22060941 (PMC12192662; doi:10.3390/ijerph22060941)

SUPPLEMENTARY MATERIAL

Table S1. List of development indicators included in the initial dataset

**Indicator [World Bank ID]**
Access to electricity (% of population) [EG.ELC.ACCS.ZS]
Adjusted net enrollment rate, primary (% of primary school age children) [SE.PRM.TENR]
Adolescent fertility rate (births per 1,000 women ages 15-19) [SP.ADO.TFRT]
Adolescents out of school (% of lower secondary school age) [SE.SEC.UNER.LO.ZS]
Adults (ages 15+) and children (ages 0-14) newly infected with HIV [SH.HIV.INCD.TL]
Adults (ages 15-49) newly infected with HIV [SH.HIV.INCD]
Average working hours of children, study and work, ages 7-14 (hours per week) [SL.TLF.0714.SW.TM]
Average working hours of children, working only, ages 7-14 (hours per week) [SL.TLF.0714.WK.TM]
Birth rate, crude (per 1,000 people) [SP.DYN.CBRT.IN]
Births attended by skilled health staff (% of total) [SH.STA.BRTC.ZS]
Cause of death, by communicable diseases and maternal, prenatal and nutrition conditions (% of total) [SH.DTH.COMM.ZS]
Cause of death, by injury (% of total) [SH.DTH.INJR.ZS]
Cause of death, by non-communicable diseases (% of total) [SH.DTH.NCOM.ZS]
Children in employment, study and work (% of children in employment, ages 7-14) [SL.TLF.0714.SW.ZS]
Children in employment, total (% of children ages 7-14) [SL.TLF.0714.ZS]
Children out of school (% of primary school age) [SE.PRM.UNER.ZS]
Community health workers (per 1,000 people) [SH.MED.CMHW.P3]
Compulsory education, duration (years) [SE.COM.DURS]
Control of Corruption: Estimate [CC.EST]
Coverage of social insurance programs (% of population) [per_si_allsi.cov_pop_tot]
Coverage of social protection and labor programs (% of population) [per_allsp.cov_pop_tot]
Coverage of social safety net programs (% of population) [per_sa_allsa.cov_pop_tot]
Coverage of unemployment benefits and ALMP (% of population) [per_lm_alllm.cov_pop_tot]
Current education expenditure, primary (% of total expenditure in primary public institutions) [SE.XPD.CPRM.ZS]
Current education expenditure, secondary (% of total expenditure in secondary public institutions) [SE.XPD.CSEC.ZS]
Current education expenditure, tertiary (% of total expenditure in tertiary public institutions) [SE.XPD.CTER.ZS]
Current education expenditure, total (% of total expenditure in public institutions) [SE.XPD.CTOT.ZS]
Current health expenditure (% of GDP) [SH.XPD.CHEX.GD.ZS]
Death rate, crude (per 1,000 people) [SP.DYN.CDRT.IN]
Domestic general government health expenditure (% of current health expenditure) [SH.XPD.GHED.CH.ZS]
Domestic general government health expenditure (% of GDP) [SH.XPD.GHED.GD.ZS]
Domestic general government health expenditure (% of general government expenditure) [SH.XPD.GHED.GE.ZS]
Domestic private health expenditure (% of current health expenditure) [SH.XPD.PVTD.CH.ZS]
Domestic private health expenditure per capita (current US$) [SH.XPD.PVTD.PC.CD]
Domestic private health expenditure per capita, PPP (current international $) [SH.XPD.PVTD.PP.CD]
Educational attainment, at least Bachelor's or equivalent, population 25+, total (%) (cumulative) [SE.TER.CUAT.BA.ZS]
Educational attainment, at least completed lower secondary, population 25+, total (%) (cumulative) [SE.SEC.CUAT.LO.ZS]
Educational attainment, at least completed post-secondary, population 25+, total (%) (cumulative) [SE.SEC.CUAT.PO.ZS]
Educational attainment, at least completed primary, population 25+ years, total (%) (cumulative) [SE.PRM.CUAT.ZS]
Educational attainment, at least completed short-cycle tertiary, population 25+, total (%) (cumulative) [SE.TER.CUAT.ST.ZS]
Educational attainment, at least completed upper secondary, population 25+, total (%) (cumulative) [SE.SEC.CUAT.UP.ZS]
Educational attainment, at least Master's or equivalent, population 25+, total (%) (cumulative) [SE.TER.CUAT.MS.ZS]
Educational attainment, Doctoral or equivalent, population 25+, total (%) (cumulative) [SE.TER.CUAT.DO.ZS]
Electric power consumption (kWh per capita) [EG.USE.ELEC.KH.PC]
Employers, total (% of total employment) (modeled ILO estimate) [SL.EMP.MPYR.ZS]
Employment in agriculture (% of total employment) (modeled ILO estimate) [SL.AGR.EMPL.ZS]
Employment in industry (% of total employment) (modeled ILO estimate) [SL.IND.EMPL.ZS]
Employment in services (% of total employment) (modeled ILO estimate) [SL.SRV.EMPL.ZS]
Employment to population ratio, 15+, total (%) (modeled ILO estimate) [SL.EMP.TOTL.SP.ZS]
Employment to population ratio, 15+, total (%) (national estimate) [SL.EMP.TOTL.SP.NE.ZS]
Exclusive breastfeeding (% of children under 6 months) [SH.STA.BFED.ZS]
Expenditure on primary education (% of government expenditure on education) [SE.XPD.PRIM.ZS]
Expenditure on secondary education (% of government expenditure on education) [SE.XPD.SECO.ZS]
Expenditure on tertiary education (% of government expenditure on education) [SE.XPD.TERT.ZS]
Fertility rate, total (births per woman) [SP.DYN.TFRT.IN]

Fixed broadband subscriptions (per 100 people) [IT.NET.BBND.P2]
Fixed telephone subscriptions (per 100 people) [IT.MLT.MAIN.P2]
GDP growth (annual %) [NY.GDP.MKTP.KD.ZG]
GDP per capita (current US$) [NY.GDP.PCAP.CD]
GDP per capita growth (annual %) [NY.GDP.PCAP.KD.ZG]
Gini index [SI.POV.GINI]
GNI (current US$) [NY.GNP.MKTP.CD]
GNI growth (annual %) [NY.GNP.MKTP.KD.ZG]
GNI per capita growth (annual %) [NY.GNP.PCAP.KD.ZG]
Goods and services expense (% of expense) [GC.XPN.GSRV.ZS]
Government Effectiveness: Estimate [GE.EST]
Government expenditure on education, total (% of GDP) [SE.XPD.TOTL.GD.ZS]
Government expenditure on education, total (% of government expenditure) [SE.XPD.TOTL.GB.ZS]
Hospital beds (per 1,000 people) [SH.MED.BEDS.ZS]
Human capital index (HCI) (scale 0-1) [HD.HCI.OVRL]
Immunization, HepB3 (% of one-year-old children) [SH.IMM.HEPB]
Incidence of HIV, all (per 1,000 uninfected population) [SH.HIV.INCD.TL.P3]
Incidence of malaria (per 1,000 population at risk) [SH.MLR.INCD.P3]
Incidence of tuberculosis (per 100,000 people) [SH.TBS.INCD]
Individuals using the Internet (% of population) [IT.NET.USER.ZS]
Land area (sq. km) [AG.LND.TOTL.K2]
Learning poverty: Share of Children at the End-of-Primary age below minimum reading proficiency adjusted by Out-of-School Children (%) [SE.LPV.PRIM]
Life expectancy at birth, total (years) [SP.DYN.LE00.IN]
Literacy rate, adult total (% of people ages 15 and above) [SE.ADT.LITR.ZS]
Literacy rate, youth (ages 15-24), gender parity index (GPI) [SE.ADT.1524.LT.FM.ZS]
Low-birthweight babies (% of births) [SH.STA.BRTW.ZS]
Maternal mortality ratio (modeled estimate, per 100,000 live births) [SH.STA.MMRT]
Maternal mortality ratio (national estimate, per 100,000 live births) [SH.STA.MMRT.NE]
Mobile cellular subscriptions (per 100 people) [IT.CEL.SETS.P2]
Mortality rate attributed to unsafe water, unsafe sanitation and lack of hygiene (per 100,000 population) [SH.STA.WASH.P5]
Mortality rate attributed to unintentional poisoning, male (per 100,000 male population) [SH.STA.POIS.P5.MA]
Mortality rate attributed to household and ambient air pollution, age-standardized (per 100,000 population) [SH.STA.AIRP.P5]
Mortality from CVD, cancer, diabetes or CRD between exact ages 30 and 70 (%) [SH.DYN.NCOM.ZS]
Mortality caused by road traffic injury (per 100,000 population) [SH.STA.TRAF.P5]
Mortality rate, infant (per 1,000 live births) [SP.DYN.IMRT.IN]
Mortality rate, neonatal (per 1,000 live births) [SH.DYN.NMRT]
Mortality rate, under-5 (per 1,000 live births) [SH.DYN.MORT]
Nurses and midwives (per 1,000 people) [SH.MED.NUMW.P3]
Number of surgical procedures (per 100,000 population) [SH.SGR.PROC.P5]
Over-age students, primary (% of enrollment) [SE.PRM.OENR.ZS]
Part time employment, total (% of total employment) [SL.TLF.PART.ZS]
People using at least basic drinking water services (% of population) [SH.H2O.BASW.ZS]
People practicing open defecation (% of population) [SH.STA.ODFC.ZS]
People using at least basic sanitation services (% of population) [SH.STA.BASS.ZS]
People using safely managed drinking water services (% of population) [SH.H2O.SMDW.ZS]
People using safely managed sanitation services (% of population) [SH.STA.SMSS.ZS]
People with basic handwashing facilities including soap and water (% of population) [SH.STA.HYGN.ZS]
Physicians (per 1,000 people) [SH.MED.PHYS.ZS]
Political Stability and Absence of Violence/Terrorism: Estimate [PV.EST]
Population density (people per sq. km of land area) [EN.POP.DNST]
Population growth (annual %) [SP.POP.GROW]
Population, total [SP.POP.TOTL]
Pregnant women receiving prenatal care (%) [SH.STA.ANVC.ZS]
Prevalence of anemia among children (% of children ages 6-59 months) [SH.ANM.CHLD.ZS]
Prevalence of anemia among non-pregnant women (% of women ages 15-49) [SH.ANM.NPRG.ZS]
Prevalence of anemia among pregnant women (%) [SH.PRG.ANEM]
Prevalence of anemia among women of reproductive age (% of women ages 15-49) [SH.ANM.ALLW.ZS]
Prevalence of current tobacco use (% of adults) [SH.PRV.SMOK]
Prevalence of HIV, total (% of population ages 15-49) [SH.DYN.AIDS.ZS]

Prevalence of moderate or severe food insecurity in the population (%) [SN.ITK.MSFI.ZS]
Prevalence of overweight (modeled estimate, % of children under 5) [SH.STA.OWGH.ME.ZS]
Prevalence of overweight, weight for height (% of children under 5) [SH.STA.OWGH.ZS]
Prevalence of severe food insecurity in the population (%) [SN.ITK.SVFI.ZS]
Prevalence of severe wasting, weight for height (% of children under 5) [SH.SVR.WAST.ZS]
Prevalence of stunting, height for age (% of children under 5) [SH.STA.STNT.ZS]
Prevalence of stunting, height for age (modeled estimate, % of children under 5) [SH.STA.STNT.ME.ZS]
Prevalence of undernourishment (% of population) [SN.ITK.DEFC.ZS]
Prevalence of underweight, weight for age (% of children under 5) [SH.STA.MALN.ZS]
Prevalence of wasting, weight for height (% of children under 5) [SH.STA.WAST.ZS]
Primary completion rate, total (% of relevant age group) [SE.PRM.CMPT.ZS]
Primary school age children out-of-school (%) [SE.LPV.PRIM.SD]
Progression to secondary school (%) [SE.SEC.PROG.ZS]
Proportion of people living below 50 percent of median income (%) [SI.DST.50MD]
Proportion of population spending more than 10% of household consumption or income on out-of-pocket health care expenditure (%) [SH.UHC.OOPC.10.ZS]
Proportion of population spending more than 25% of household consumption or income on out-of-pocket health care expenditure (%) [SH.UHC.OOPC.25.ZS]
Proportion of seats held by women in national parliaments (%) [SG.GEN.PARL.ZS]
Pupil-teacher ratio, lower secondary [SE.SEC.ENRL.LO.TC.ZS]
Pupil-teacher ratio, preprimary [SE.PRE.ENRL.TC.ZS]
Pupil-teacher ratio, primary [SE.PRM.ENRL.TC.ZS]
Pupil-teacher ratio, secondary [SE.SEC.ENRL.TC.ZS]
Pupil-teacher ratio, tertiary [SE.TER.ENRL.TC.ZS]
Pupil-teacher ratio, upper secondary [SE.SEC.ENRL.UP.TC.ZS]
Ratio of female to male labor force participation rate (%) (modeled ILO estimate) [SL.TLF.CACT.FM.ZS]
Ratio of female to male labor force participation rate (%) (national estimate) [SL.TLF.CACT.FM.NE.ZS]
Regulatory Quality: Estimate [RQ.EST]
Research and development expenditure (% of GDP) [GB.XPD.RSDV.GD.ZS]
Rule of Law: Estimate [RL.EST]
Rural land area (sq. km) [AG.LND.TOTL.RU.K2]
Rural population (% of total population) [SP.RUR.TOTL.ZS]
School enrollment, preprimary (% gross) [SE.PRE.ENRR]
School enrollment, primary (% gross) [SE.PRM.ENRR]
School enrollment, secondary (% gross) [SE.SEC.ENRR]
School enrollment, tertiary (% gross) [SE.TER.ENRR]
Total alcohol consumption per capita (liters of pure alcohol, projected estimates, 15+ years of age) [SH.ALC.PCAP.LI]
Unemployment with advanced education (% of total labor force with advanced education) [SL.UEM.ADVN.ZS]
Unemployment with basic education (% of total labor force with basic education) [SL.UEM.BASC.ZS]
Unemployment with intermediate education (% of total labor force with intermediate education) [SL.UEM.INTM.ZS]
Unemployment, total (% of total labor force) (modeled ILO estimate) [SL.UEM.TOTL.ZS]
Unemployment, total (% of total labor force) (national estimate) [SL.UEM.TOTL.NE.ZS]
Urban population (% of total population) [SP.URB.TOTL.IN.ZS]
Urban population growth (annual %) [SP.URB.GROW]
Voice and Accountability: Estimate [VA.EST]
Women Business and the Law Index Score (scale 1-100) [SG.LAW.INDX]

Table S2. Summary of missing values before imputation

Indicator	Proportion of missing
Fixed broadband subscriptions (per 100 people)	19.1%
Women Business and the Law Index Score (scale 1-100)	16.9%
Prevalence of stunting, height for age (modeled estimate, % of children under 5)	14.8%
Prevalence of overweight (modeled estimate, % of children under 5)	13.7%
School enrollment, primary (% gross)	12.8%
Immunization, HepB3 (% of one-year-old children)	12.7%
Incidence of tuberculosis (per 100,000 people)	12.5%
Compulsory education, duration (years)	8.7%
Maternal mortality ratio (modeled estimate, per 100,000 live births)	8.1%
Domestic private health expenditure per capita, PPP (current international $)	7.5%
Domestic general government health expenditure (% of current health expenditure)	7.5%
Domestic private health expenditure (% of current health expenditure)	7.5%
Domestic private health expenditure per capita (current US$)	7.5%
Domestic general government health expenditure (% of GDP)	7.4%
Current health expenditure (% of GDP)	7.2%
Proportion of seats held by women in national parliaments (%)	4.7%
Employers, total (% of total employment) (modeled ILO estimate)	4.0%
Employment in agriculture (% of total employment) (modeled ILO estimate)	4.0%
Employment in industry (% of total employment) (modeled ILO estimate)	4.0%
Employment in services (% of total employment) (modeled ILO estimate)	4.0%
Employment to population ratio, 15+, total (%) (modeled ILO estimate)	4.0%
Ratio of female to male labor force participation rate (%) (modeled ILO estimate)	4.0%
Unemployment, total (% of total labor force) (modeled ILO estimate)	4.0%
Individuals using the Internet (% of population)	2.7%
People practicing open defecation (% of population)	2.5%
GNI (current US$)	2.2%
People using at least basic drinking water services (% of population)	2.0%
Fixed telephone subscriptions (per 100 people)	1.5%
GDP per capita growth (annual %)	1.2%
GDP growth (annual %)	1.1%
People using at least basic sanitation services (% of population)	1.1%
GDP per capita (current US$)	0.6%
Mobile cellular subscriptions (per 100 people)	0.6%
Life expectancy at birth, total (years)	0.4%
Fertility rate, total (births per woman)	0.3%
Access to electricity (% of population)	0.2%
Land area (sq. km)	0.2%
Population density (people per sq. km of land area)	0.2%

Table S3. Summary statistics of development indicators included in the analysis

Indicator	Mean	Std Dev	Median	IQR
Immunization, measles (% of children ages 12-23 months)	84.51	14.9	90	19
Immunization, DPT (% of children ages 12-23 months)	85.35	14.71	91	17
Access to electricity (% of population)	78.54	29.13	95.89	39.74
Adolescent fertility rate (births per 1,000 women ages 15-19)	55.91	42.58	45.44	61.53
Birth rate, crude (per 1,000 people)	22.36	10.79	20.42	18.16
Compulsory education, duration (years)	9.31	2.12	9	2
Current health expenditure (% of GDP)	6.39	2.84	5.61	3.67
Death rate, crude (per 1,000 people)	8.38	3.1	7.8	3.61
Domestic general government health expenditure (% of current health expenditure)	50.04	21.04	50.27	32.84
Domestic general government health expenditure (% of GDP)	3.28	2.3	2.65	2.92
Domestic private health expenditure (% of current health expenditure)	42.57	18.67	43.15	27.72
Domestic private health expenditure per capita (current US$)	285.47	581.05	87.68	240.83
Domestic private health expenditure per capita, PPP (current international $)	419.72	571.51	225.39	475.73
Employers, total (% of total employment) (modeled ILO estimate)	3.36	2.16	3.19	2.67
Employment in agriculture (% of total employment) (modeled ILO estimate)	28.59	22.09	24.33	36.09
Employment in industry (% of total employment) (modeled ILO estimate)	19.75	7.85	20.32	10.67
Employment in services (% of total employment) (modeled ILO estimate)	51.66	17.66	52.13	28.02
Employment to population ratio, 15+, total (%) (modeled ILO estimate)	56.06	10.87	56.35	12.5
Fertility rate, total (births per woman)	2.96	1.48	2.5	2.26
Fixed broadband subscriptions (per 100 people)	8.2	11.48	1.96	12.36
Fixed telephone subscriptions (per 100 people)	16.21	16.57	11.42	21.62
GDP growth (annual %)	3.47	4.93	3.74	4.06
GDP per capita (current US$)	10667.24	16181.24	3967.2	10193.67
GDP per capita growth (annual %)	2.05	4.87	2.2	3.87
GNI (current US$)	2.33E+12	7.99E+12	4.92E+10	5.39E+11
Incidence of tuberculosis (per 100,000 people)	131.99	178.63	55	175
Individuals using the Internet (% of population)	35.1	30.86	27	56.41
Land area (sq. km)	5843567	15850730	298170	1852555
Life expectancy at birth, total (years)	69.5	8.56	71.19	12.03
Maternal mortality ratio (modeled estimate, per 100,000 live births)	198.44	250.89	83	254
Mobile cellular subscriptions (per 100 people)	71.32	49.29	76.22	90.41
Mortality rate, infant (per 1,000 live births)	29.17	25.5	20.9	37.07
Mortality rate, neonatal (per 1,000 live births)	16.5	12.55	13.08	21
Mortality rate, under-5 (per 1,000 live births)	40.8	40.94	24.7	52.3
People using at least basic drinking water services (% of population)	84.17	17.96	91.83	23.54
People practicing open defecation (% of population)	10.64	16.17	2.33	16.02
People using at least basic sanitation services (% of population)	70.62	29.24	83.36	50.23
Population density (people per sq. km of land area)	158.55	500.95	65.79	99.67
Population growth (annual %)	1.38	1.34	1.29	1.77
Population, total	3.23E+08	9.5E+08	11933041	80008962
Prevalence of overweight (modeled estimate, % of children under 5)	6.85	4.09	6.1	5.1
Prevalence of stunting, height for age (modeled estimate, % of children under 5)	21.21	14.28	18.6	24.3
Proportion of seats held by women in national parliaments (%)	18.5	10.92	17.33	14.46
Ratio of female to male labor force participation rate (%) (modeled ILO estimate)	69.23	19.44	74.17	22.31
Rural population (% of total population)	44.58	21.73	44.52	36.48

School enrollment, primary (% gross)	100.93	12.64	101.29	9.19
Unemployment, total (% of total labor force) (modeled ILO estimate)	7.92	5.57	6.37	5.88
Urban population (% of total population)	55.42	21.73	55.48	36.49
Urban population growth (annual %)	2.14	1.77	2.08	2.44
Women Business and the Law Index Score (scale 1-100)	70.22	18.01	73.13	25.42

Table S5. Correlation, association, and agreement between PC1 score and immunization coverage across years

Year	Measles			DPT		
	Spearman correlation coefficient	Chi-square p-value	Weighted Kappa	Spearman correlation coefficient	Chi-square p-value	Weighted Kappa
2016	-0.55	< 0.0001	0.52	-0.55	< 0.0001	0.56
2017	-0.59	< 0.0001	0.57	-0.59	< 0.0001	0.57
2018	-0.60	< 0.0001	0.57	-0.60	< 0.0001	0.55
2019	-0.58	< 0.0001	0.57	-0.58	< 0.0001	0.55
2020	-0.62	< 0.0001	0.54	-0.62	< 0.0001	0.54
2021	-0.61	< 0.0001	0.56	-0.61	< 0.0001	0.52

Table S6. List of countries according to PC1 score quartile distribution in 2021

Quartile	Countries
**Q1**	Luxembourg; Norway; Denmark; Korea, Rep.; Singapore; Switzerland; United States; Germany; Ireland; Malta; Finland; Iceland; Greece; Japan; Netherlands; United Kingdom; France; Qatar; Kuwait; Estonia; Australia; New Zealand; Czechia; Canada; Sweden; Saudi Arabia; Italy; Oman; Spain; Cyprus; Lithuania; Latvia; Belgium; Montenegro; Russian Federation; Chile; Uruguay; Portugal; Lebanon; Iran, Islamic Rep.; Austria; Bahrain; Belarus
**Q2**	Türkiye; Poland; Argentina; Costa Rica; Mexico; El Salvador; Serbia; Cuba; Dominican Republic; Ukraine; The Bahamas; Colombia; Trinidad and Tobago; Libya; Peru; Moldova; Paraguay; Barbados; Tunisia; Bosnia and Herzegovina; Ecuador; Thailand; Mongolia; Armenia; Guyana; Iraq; Guatemala; Venezuela, RB; Albania; Suriname; St. Vincent and the Grenadines; Fiji; Morocco; Egypt, Arab Rep.; West Bank and Gaza; Kyrgyz Republic; Nicaragua; Uzbekistan; Algeria; Azerbaijan; Sri Lanka
**Q3**	Djibouti; Tonga; Syrian Arab Republic; Turkmenistan; Gabon; Indonesia; Belize; South Africa; Honduras; Philippines; Bolivia; Botswana; Bhutan; Tajikistan; Lao PDR; Samoa; Sao Tome and Principe; Namibia; Nepal; Pakistan; Gambia; Comoros; Equatorial Guinea.
**Q4**	Haiti; Mauritania; Senegal; Kenya; Vanuatu; Congo, Rep.; Timor-Leste; Lesotho; Solomon Islands; Sudan; Rwanda; Cote d'Ivoire; Afghanistan; Guinea-Bissau; Papua New Guinea; Yemen, Rep.; Nigeria; Liberia; Guinea; Togo; Malawi; Cameroon; Ethiopia; Mali; Zimbabwe; Madagascar; Tanzania; Mozambique; Somalia; Uganda; Zambia; Sierra Leone; Benin; Angola; South Sudan; Chad; Burkina Faso; Burundi; Central African Republic; Congo, Dem. Rep.; Niger

Figure S1. Heatmap of Spearman's Correlations between 48 Socio-Economic Indicators and Immunization Coverage. Each cell represents the correlation between one indicator and one of the immunization measures. Significance levels are denoted by asterisks: * $p < 0.05$, ** $p < 0.01$, and *** $p < 0.001$.

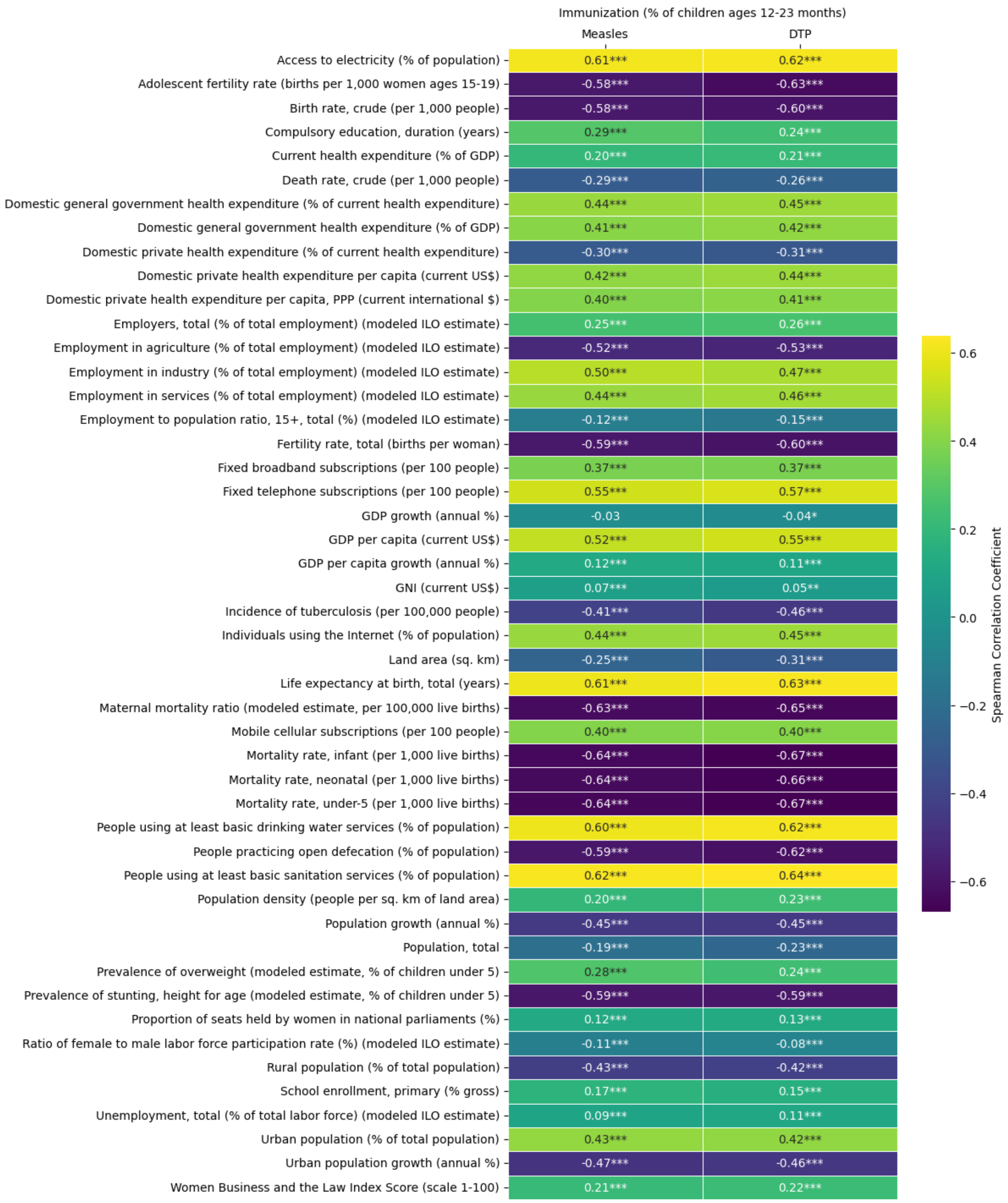

Figure S2. Variance explained by principal components

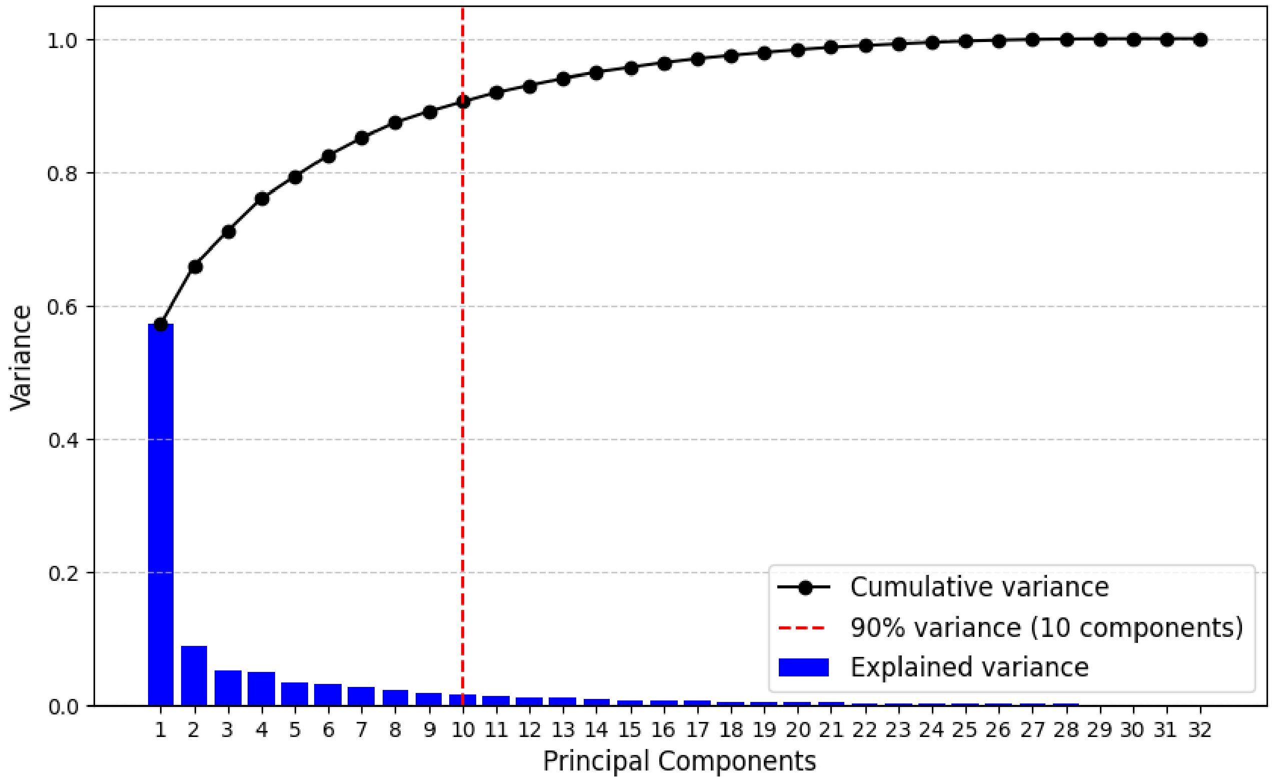

Figure S3. Scree plot

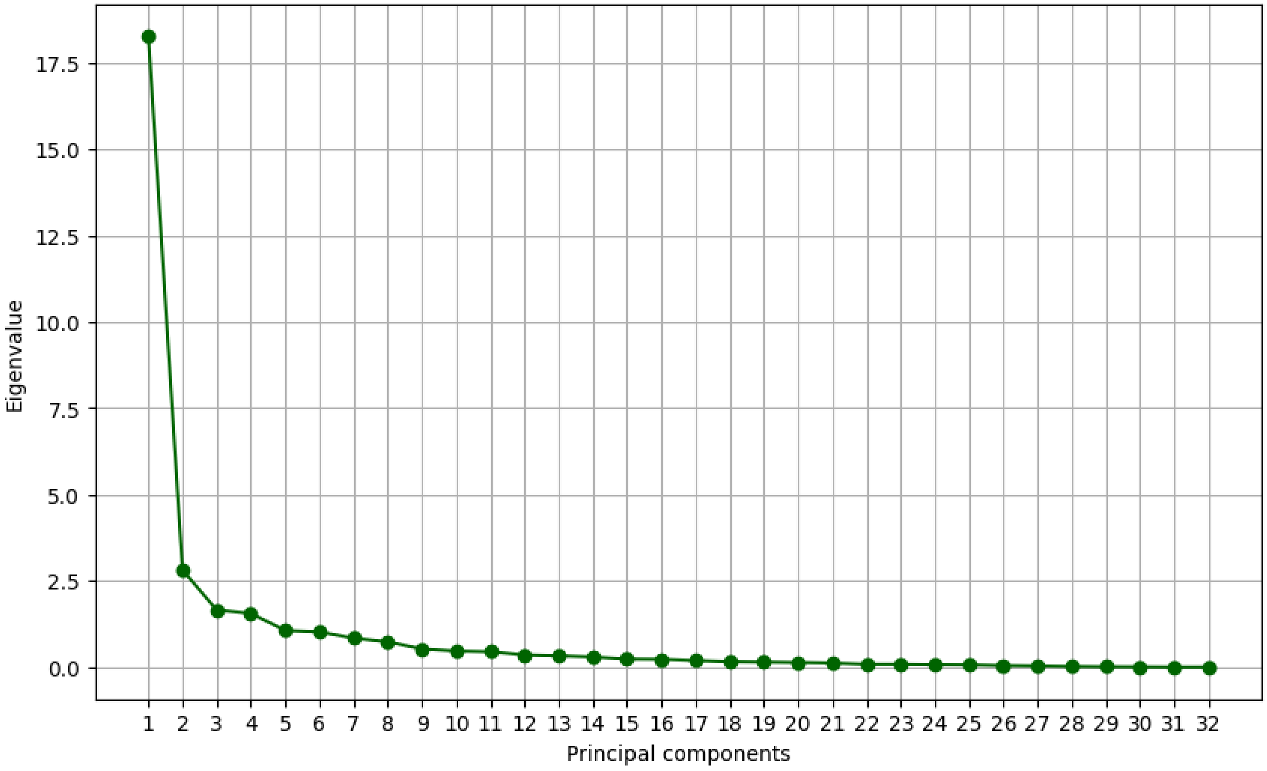

Figure S4. Heatmap showing factors loadings for each indicator and principal component

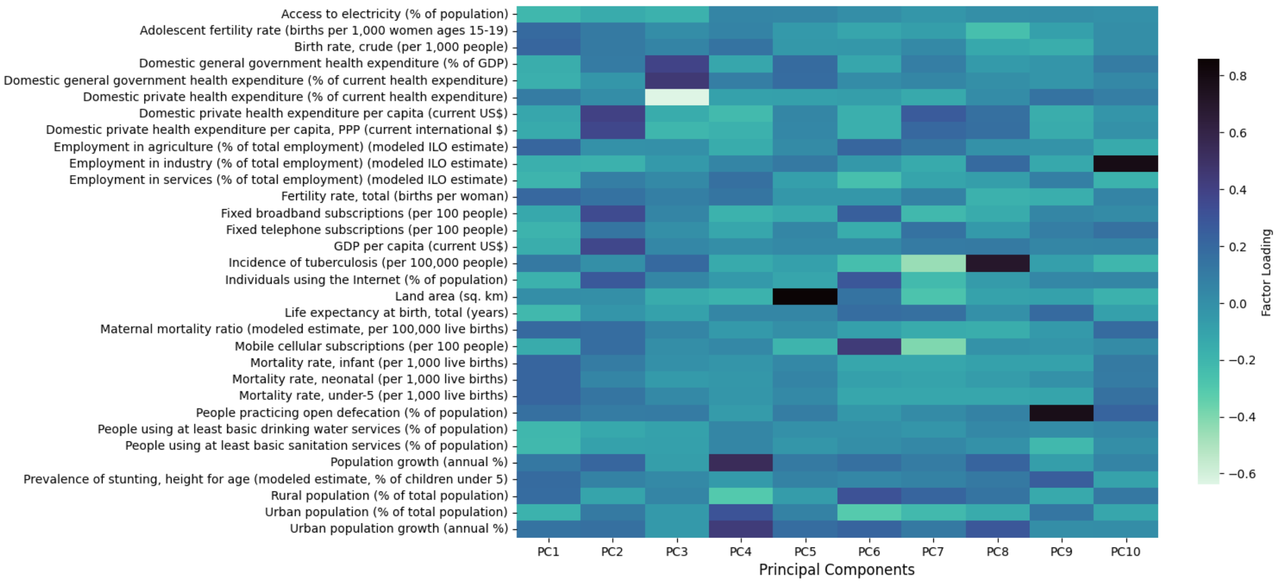

Supplement: Supplementary file 1 [file ijerph-22-00941-s001.zip › Supplementary File 1.pdf]
